# Maternal and Perinatal Outcomes of Exercise in Pregnant Women with Chronic Hypertension and/or Previous Preeclampsia: A Randomized Controlled Trial

**DOI:** 10.1155/2013/857047

**Published:** 2013-08-12

**Authors:** Karina Tamy Kasawara, Camila Schneider Gannuny Burgos, Simony Lira do Nascimento, Néville Oliveira Ferreira, Fernanda Garanhani Surita, João Luiz Pinto e Silva

**Affiliations:** 101 Alexander Fleming Avenue, Cidade Universitária Zeferino Vaz, 13083-881 Campinas, SP, Brazil

## Abstract

*Objectives*. To evaluate the association between physical exercise supervised in pregnant women with chronic hypertension and/or previous preeclampsia and maternal and neonatal outcomes. *Method*. Randomized controlled trial, which included 116 pregnant women with chronic hypertension and/or previous preeclampsia, considered risk of preeclampsia development. They were divided into two groups: study group that performed physical exercise with a stationary bicycle once a week, for 30 minutes; the intensity was controlled (heart rate 20% above resting values), under professional supervision and a control group that was not engaged in any physical exercise. The data was retrieved from medical charts. Significance level assumed was 5%. *Results*. Women from study group performed 9.24 ± 7.03 of physical exercise sessions. There were no differences between groups comparing type of delivery and maternal outcomes, including maternal morbidity and hospitalization in intensive unit care, and neonatal outcomes, including birth weight, adequacy of weight to gestational age, prematurity, Apgar scale at first and fifth minutes, hospitalization in intensive unit care, and neonatal morbidity. *Conclusions*. Physical exercise using a stationary bicycle in pregnant women with chronic hypertension and/or previous preeclampsia, once a week, under professional supervision, did not interfere in the delivery method and did not produce maternal and neonatal risks of the occurrence of morbidity. This trial is registered with ClinicalTrials.gov NCT01395342.

## 1. Introduction

Hypertensive disorders during pregnancy are one important cause of maternal deaths worldwide, particularly in developing countries. Hypertensive disorders are responsible for 26% of maternal deaths in Latin America and in the Caribbean, in comparison to 16% in developed countries [1]. A study carried out in all states of Brazil indicated that hypertensive disorders are a leading source of mortality, accounting for about 25% of maternal deaths in Brazil [2]. Despite a reduction of maternal mortality in Brazil, these rates are still high. 

It is well known that pregnant women with chronic hypertension (CH), or those who already had preeclampsia (PE) in previous pregnancies, have an increased recurrence risk of PE in subsequent pregnancies and have several other related clinical and obstetrical complications [3–5]. Among these complications are a higher probability of repeat PE, higher rates of operative deliveries, maternal and neonatal admission to intensive therapy units (ICU) [6], high rate of lower gestational age, and low birth weight [3, 7].

Rest is usually recommended to prevent morbidity for these pregnant women. However, there is insufficient scientific evidence to recommend systematic rest, as a method to prevent PE development and its complications [8].

Exercise and physical activity are associated with a reduced risk and the necessity of medication for treatment of hypertension in nonpregnant subject [9]. The practice of physical exercise is also recommended in a no-risk and/or low-risk pregnancy because of its benefits to maternal health. In addition, exercise is considered a safe activity for both, mother and the fetus, especially when performed under professional guidance and supervision [10–12]. In low-risk pregnant women, when low-intensity and moderate-intensity exercise is performed, it does not alter newborn weight [10, 13–15] and reduces the risk of prematurity [16, 17]. However, there is lack of data about the effects of physical exercise during a high-risk pregnancy and its impact on the mother and newborn.

 Recently, recommendations about exercise in pregnant women with hypertension or at risk of PE development have been studied with the objective of trying to reduce the deleterious effects of hypertensive disorders in pregnancy, including the reduction of the incidence of PE [18–21]. The mechanism involved would be that PE could be prevented through physical exercise by reducing blood pressure levels and promoting better cardiovascular fitness in pregnant women [22]. In addition, exercise may decrease maternal concentration of oxidative substances, stimulate placental growth, and act on the reversal of endothelial dysfunction [23].

Physiologically, the performance of physical exercise during pregnancy offers benefits. Furthermore, it is well known that physical activity has an important role in hypertensive subject. There is a lack of knowledge about whether effects of physical exercise are associated or not with a maternal or fetal risk in pregnant women with hypertensive disorders or those with risk of developing PE. The aim of the present study was to evaluate the association between physical exercise supervised in pregnant women with CH and/or previous PE and maternal and neonatal outcomes.

This project has been presented in an oral session at “XVIII World Congress of the International Society for Study of Hypertension in Pregnancy (ISSHP)” on July 9 to 12, 2012, at Geneva, Switzerland.

## 2. Materials and Methods

A randomized controlled trial (RCT) was conducted between January 2008 and November 2011, in the Obstetrics Unit and in the Physical Therapy Unit at Outpatient Clinic of the Women's Hospital Professor Dr. José Aristodemo Pinotti (CAISM) of the University of Campinas (UNICAMP). Approval was obtained from the Research Ethics Committee of the Medical School of the University of Campinas (FCM-UNICAMP) (929/2007).

Sample size was calculated by using a comparison of prevalence rates of PE in women at higher risk of developing the disorder (16% to 18%) [24]. Considering a significance level of 5% and a power of 80%, sample size was estimated at *n* = 58 participants for each group. After that, maintaining the same level of significance, the power of the test was calculated, based on the results found for the main variables: weight of the newborn (80.3%) and Apgar score assigned at one minute (99%).

Eligible pregnant women according to preestablished criteria were selected in the prenatal outpatient clinic and invited to participate in the study.

One-hundred and sixteen pregnant women were randomized, diagnosed with CH, a history of PE in previous pregnancies or both, between 12 and 20 weeks of gestation, and aged over 18 years. CH was defined as hypertension (blood pressure ≥ 140/90 mmHg) diagnosed before pregnancy or before completing 20 weeks of gestation. Previous preeclampsia was considered a reported history of hypertension and proteinuria after 20 weeks of gestation in previous pregnancies. 

Women with multiple pregnancies, cervical insufficiency, vaginal bleeding, heart disease, systemic lupus erythematosus, kidney failure, and neurologic disorders were excluded from the study. For inclusion in the study, pregnant women also could not be engaged in any supervised physical exercise, at the time of selection and when signing a free written informed consent term. 

Pregnant women were randomized using an opaque sealed envelope that was sequentially numbered and statistically generated by a computer program, with allocation concealment (shielding the investigator). The envelope contained information about the random allocation group: a study group (SG), engaged in physical exercise with a stationary bicycle (SB) once a week under the supervision of a physical therapist, or a control group (CG), not engaged in any physical exercise. Random allocation of the subjects was performed by another investigator, who did not participate directly in the research study. 

Pregnant women from the SG performed thirty minutes of physical exercise using an SB, BM40000 Movement horizontal bench professional model, under the supervision of the investigator, once a week after study inclusion (between 12 and 20 weeks of gestation), every week until the end of pregnancy. 

The session began with adequate preparation of the pregnant woman for the performance of physical exercise. The woman was seated in a chair wearing a protective foot covers. Subsequently, BP was measured and the watch and heart rate monitor waistband (POLAR model CS300 multi) were positioned to record heart beats per minute (bpm). The watch was placed in the left wrist and the band was adapted to the inframammary region.

The seat of the SB was individually adapted for postural correction and height of the pedal according to weight, height, and gestational week. As a result, the woman remained in a comfortable position and was maximally protected from possible joint damage. Pregnant women started to cycle and were instructed to try to maintain a heart rate 20% above resting values, not surpassing a value of 140 bpm and the exercise was performed regarding American College of Obstetricians and Gynecologists guidelines [25] during the 30 minutes of exercise performance. Exercise intensity was gradually adjusted until the proposed parameters were reached. At approximately two minutes before the end of the exercise, the woman was instructed to decrease the speed of cycling, until the end of the established time. Stretching exercises were performed for five minutes following instructions, with the woman still on the bicycle, prioritizing the anterior and posterior chains of the lower limbs and relaxation of the lumbar spine. In the end, the woman was removed from the SB with the assistance of the physical therapist, seated for five minutes in a comfortable chair, and waiting for the final blood pressure measurement. 

Exercise with an SB was performed in an adequate setting, with appropriate ventilation and illumination. Pregnant women were instructed to have a light meal about one hour before the performance of exercise and to wear comfortable clothes.

Pregnant women from the CG did not receive instructions on the practice of physical exercise and followed routine prenatal care.

Data related to sociodemographic and clinical characteristics (age, BMI, race, marital status, obstetrical data, history of CH and PE, and the practice of previous physical exercise) were collected at the time the pregnant woman was included in the study. Data about delivery and maternal and neonatal outcomes were recorded from the medical charts by the researcher responsible and transcribed to a file specially designed for the collection of information. When the delivery did not happen in CAISM, additional contact was made by direct telephone call or personally to supplement possible information.

Maternal outcomes evaluated were admission to the ICU and morbid conditions defined by the presence of any complications during pregnancy, delivery, or postpartum period.

The variables considered related to neonatal outcomes were: birth weight, adequacy of weight to gestational age [26], gestational age calculated at the first trimester by ultrasound, Apgar at the first and fifth minutes, admission to the neonatal ICU, and neonatal morbidity (respiratory distress syndrome, intraventricular hemorrnage, and others). 

An intention to treat analysis was performed, without replacement values for missing data. Sociodemographic and clinical characteristics were evaluated by the chi-square tests or Fisher's exact test (for qualitative variables) and by Student's *t*-test or Mann-Whitney test (for quantitative variables), in addition to calculation of relative risk (RR) and their respective 95% confidence intervals (CI). For neonatal outcomes, a COX multiple regression model technique was used to calculate the value of risk adjusted to body mass index (BMI), race, number of pregnancies, CH and history of PE, and their respective 95% CI. SAS program version 9.2 was used for all analyses and the significance level assumed was 5%.

## 3. Results

**Figure 1:**
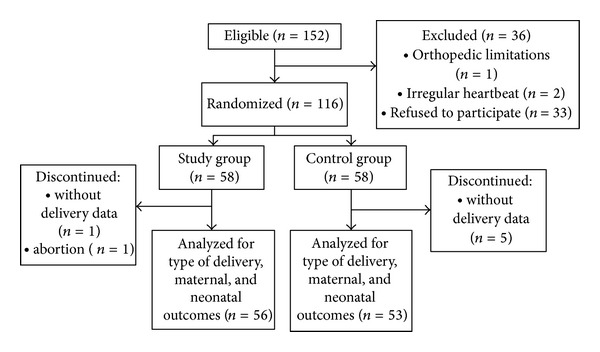
Flow chart of inclusion of pregnant women in the study.

 Among the eligible pregnant women (*n* = 152), 36 were excluded and 116 randomized. Of the randomized women, 58 were allocated to each group. Three pregnant women from the SG and nine from the CG did not give birth in CAISM and data was recorded from the charts of six of these women. One pregnant woman discontinued the study due to abortion, failing to perform any physical exercise session, since the event occurred soon after randomization (15 weeks of gestation). Fifty-six women from the SG and 53 from the CG were analyzed for the variables mode of delivery and maternal/fetal outcomes (Figure 1).

The groups were considered homogeneous in all sociodemographic and clinical variables evaluated. Most pregnant women were white, obese, led a sedentary lifestyle, aged between 30 and 39 years, and had a steady partner. Among the risk factors, 31 had PE in a previous pregnancy, 105 had CH, and 20 had both conditions combined (Table 1).

The mean number of physical exercise sessions performed by the 58 pregnant women from the SG was 9.24 ± 7.03. Of the women who performed few sessions (below the mean value of the group), 14 chose to interrupt the exercise, due to a change of city address/prenatal location. 

No complications were observed during physical exercise sessions, for example, hypertensive crisis, hypotension, hyperthermia, musculoskeletal lesions, or other complications identified that demanded interruption of the exercise. 

There were no differences between the groups regarding mode of delivery, reasons for cesarean section, and maternal complications. Among the 77 pregnant women who had cesarean sections, 23 had more than one reason. The reasons for C-section were maternal disease, repeat cesarean sections, and fetal distress. The most prevalent maternal morbidity was PE. There was one patient with the HELLP syndrome and one with acute pulmonary edema in the SG and CG, respectively (Table 2). Recurrence of PE (4.6%) was observed in two pregnant women from the SG and three from the CG (data not shown in the tables). 

There was one fetal death due to difficult-to-control hypertension in a woman with previous PE and chronic hypertension. She was hospitalized in CAISM, at 26 weeks of gestation and severe fetal growth restriction (FGR), for blood pressure and fetal vitality control. She presented an abnormal doppler velocimetry flow in umbilical artery and ductus venosus, both with reversed diastole. Fetal demise was confirmed three days after; stillbirth weight was 0.460 kg or 460 g.

The majority of newborn was of the male gender (55.5%). Mean gestational age was 38.2 ± 1.9 in the SG and 37.5 ± 2.2 in the CG (*P* = 0.09) (data not shown in the table). There were no differences in variables related to neonatal outcomes (Table 3). Among the neonatal morbidity, the most prevalent was respiratory distress syndrome (10.6%), followed by hypoglycemia (7.45%). 

After adjusted multiple regression analysis, physical exercise did not represent a risk of the neonatal outcomes studied: low birth weight (<2500 g), macrosomia (≥4000 g), adequacy of weight, and prematurity (<37 weeks of gestation) (Table 4).

## 4. Discussion

 The results of this study showed that physical exercise with an SB in pregnant women with CH and/or previous PE did not increase risk of maternal and neonatal outcomes and especially did not represent risk of hypertensive complications, prematurity, low fetal weight, or C-section rates.

This RCT was conducted in CAISM, a tertiary hospital; it is a referral center for high-risk prenatal care for the health units for this region, southeast of Brazil (city of Campinas, Brazil). This condition, added to the fact that most pregnant women previously led a sedentary lifestyle, could explain the great amount of women refusing to participate in the study and lower adherence to exercise programs or any other aerobic activity [27, 28]. Many women had difficulty in meeting the demands and proposals of the program, discontinuing after some sessions, missing many sessions, and showing a low protocol adherence.

Regarding morbidity maternal outcomes, the most prevalent in this study was PE. It was expected due to the sample characteristics, all of them had risk of development of PE. However, it could be observed that physical exercise did not increase the rate of PE development; the occurrence of PE was the same in both groups. Regardless of the number of exercise sessions, since the rate of women who did not have a morbid condition was 84.4%, these results corroborated the findings by Yeo et al. [29] who studied pregnant women with a previous history of PE and also found no difference in PE development between those engaged in walking and those performing stretching exercises, five times a week during pregnancy. 

In our study, the recurrence of PE was observed in five pregnant women (two from the SG and three from the CG), suggesting that physical activity with an SB once a week did not interfere in PE development and may be considered safe for pregnant women with previous PE. It has been well established that the recurrence of PE is associated with worse neonatal outcomes [30]. The occurrences of PE in women with CH were considered superimposed PE and they were distributed in both groups without significant difference. 

High cesarean section rates were observed in both groups (70.6%). In Brazil there is a national problem with the high rate of C-sections. However it does not justify the results in this study. 

Furthermore the high rates of cesarean section found in this study could be related to the characteristics of sample selection. In addition, the majority pregnant women enrolled in our study were obese and there was a high rate of repeat C-section. 

It is well known that obesity is an important risk factor for operative delivery [31]. In a meta-analysis including 33 studies, overweight, obese, and morbidly obese pregnant women had a twofold to threefold increased risk of cesarean section, when compared to pregnant women of normal weight. The risk increased proportionally to an increase in BMI in overweight (OR = 1.46, 95% CI 1.34–1.60), obese (OR = 2.05, 95% CI 1.86–2.27), and morbidly obese (OR = 2.89, 95% CI 2.28–3.79) pregnant women [32].

There is no consensus in the literature about an association between preterm delivery in low-risk pregnant women and physical [33–35] or occupational [16, 36, 37] activity during pregnancy. As in our study there was no risk of prematurity in the SG. This corroborated a Cochrane review that also demonstrated a lack of association between aerobic exercise and prematurity in low-risk pregnant women (RR = 1.82, 95% CI 0.35–9.57) [38]. Another study conducted in the Brazil (south of the country) demonstrated that leisure time activity during pregnancy was associated with a lower risk of prematurity [39].

We found only one study on physical activity (walking versus stretching) in pregnant women at risk of previous PE. That study demonstrated a prematurity rate of 22% among pregnant women who walked compared to 11% who only performed stretching exercises (no significant difference) [29]. An explanation for the different results obtained could be that the controls were distinct (stretching versus walking), as well as the intervention performed (no intervention versus SB). Furthermore, physical exercise sessions for our patients took place under direct and continuous professional supervision, permitting better control of the intensity and uniformity of the programmed activity and promoting better results. 

The incidence of low birth weight (<2500 g) was not different in both groups. These results are similar to those in a study by Yeo et al. [29] who also evaluated pregnant women at risk, in which no difference in newborn weight was observed among women who walked and stretched throughout pregnancy. 

There is a controversy about exercise performed in the first trimester and birth weight. Some researchers observed that women who started moderate-intensity physical exercise in the first trimester [40, 41] or who had an occupational activity [42] had infants with a lower birth weight. However, there is a study showing that sedentary women had newborns with a lower birth weight [37]. The physiological changes were observed by Clapp et al. [40] who found an increase in the velocity of placental growth and improvement in placental function which could be attributed to favorable physiological alterations due to physical exercise, such as an increase in maternal blood circulation.

In contrast, women performing high-intensity physical activity during pregnancy may have more low-weight and small-for-gestational-age (SGA) newborns at birth [33, 43]. These neonatal outcomes are also caused by the presence of CH, which is known to increase the risk of SGA and low Apgar scores at one and five minutes [3]. In our study, there was no difference in adequacy of weight to gestational age, as well as in Apgar scores, probably because exercise was of low intensity and controlled, therefore considered safe for fetal vitality. 

A recent RCT evaluating the effect of aerobic exercise on sedentary pregnant women who danced or stretched during 60-minute periods, twice a week, and exercised 30 additional minutes at home in alternate days showed better and higher 1-minute Apgar scores in the exercise group, without any significant difference at five minutes of life [15]. It is well known that a low Apgar score, especially one that persists at five minutes, is indicative of higher neonatal mortality and morbidity [44, 45]. In the present study, although Apgar scores showed no difference in both time periods measured, neonatal morbidity rate was equally elevated in both groups. This could be related to a greater presence of premature infants and low birth weight.

In a recent systematic review of randomized controlled trial, case control and cohort studies regarding exercise and physical activity in the prevention of PE performed by health pregnant women showed a possible protective effect of leisure time physical activity in the development of PE [46]. 

However the American College of Obstetrics and Gynecology (ACOG) considered pregnant women and those with chronic arterial hypertension as relative contraindications for physical exercise [25]. Because of that, we decided to develop the program with low-intensity systematized physical exercise performed only once a week after medical permission, under the direct and continuous supervision of a specialized trained physical therapist, in a hospital-based outpatient facility specialized in high-complexity care of pregnant women, with continuous medical supervision. In case of any complication, additional support was readily available.

Women from the SG permitted the observation of the physiological effects of physical exercise performed during pregnancy on maternal well-being, in addition to establishing a closer link to healthcare professionals who participated in the prenatal care in weekly meetings. It may be speculated that women who understood the importance of preventing PE and other complications of pregnancy were those who best adhered to the exercise program with an SB.

## 5. Conclusions

 In conclusion, physical exercise using an SB in pregnant women with CH and/or previous PE, performed once a week under professional supervision, did not produce maternal and neonatal risk. The physical exercise was safe and it was not harmful to mother and newborn. This study may encourage other clinical trials with higher frequency and duration of exercise sessions. Women with CH and/or previous PE, even those leading a sedentary lifestyle before pregnancy, may initiate physical exercise with controlled intensity and adequate prescription. In future studies, it may be relevant to evaluate adherence to physical exercise and lifestyle changes begun during the gestational period and the benefits of perpetuating this highly recommended behavior in these women. Pregnancy determines individual and familial mobilization; it could represent a particularly opportune moment to initiate lifestyle changes in women with hypertensive disorders or at risk of this morbidity condition. 

## Figures and Tables

**Table 1:** Baseline characteristics of pregnant women with chronic hypertension and/or previous PE, according to exercise or nonexercise group.

Variable	Study group (*n* = 58)	Control group (*n* = 58)	*P* value^a^
Age (years), *n* (%)			0.82^b^
<19	1 (1.7)	1 (1.7)	
20–29	21 (36.2)	20 (34.5)	
30–39	27 (46.6)	31 (53.5)	
≥40	9 (15.5)	6 (10.3)	
BMI at inclusion, kg/m²			0.57
18.5–24.9	4 (6.9)	6 (10.3)	
25–29.9	13 (22.4)	11 (19.0)	
30–39.9	26 (44.8)	31 (53.5)	
≥40	15 (25.9)	10 (17.2)	
Race/color, *n* (%)			0.24
Nonwhite	17 (29.3)	23 (39.7)	
Mean of gestational age at inclusion^d^	17.3 ± 3.4	18.5 ± 3.4	0.62^c^
Parity, *n* (%)			0.34
0	13 (22.4)	9 (15.5)	
≥1	45 (77.6)	49 (84.5)	
Previous abortion, *n* (%)	17 (29.3)	15 (25.9)	0.67
Previous PE, *n* (%)	16 (27.6)	15 (25.9)	0.83
CH, *n* (%)	51 (87.9)	54 (93.1)	0.34
CH and previous PE, *n* (%)	9 (15.5)	11 (19)	0.62
Marital status, *n* (%)			1.00^b^
With a partner	53 (91.4)	54 (93.1)	
Physical exercise prior pregnancy, *n* (%)	6 (10.5)	4 (7.1)	0.74^b^

^a^Calculated with chi-square test; ^b^calculated with Fisher's exact test. ^c^Calculated with Student's *t*-test; ^d^data are given as mean ± SD.

CH: chronic hypertension, BMI: body mass index, and PE: preeclampsia.

**Table 2:** Mode of delivery and maternal outcomes in pregnant women with chronic hypertension and/or previous PE, according to exercise or nonexercise group.

Outcome	Study group (*n* = 56)	Control group (*n* = 53)	Relative risk (95% CI)	*P* value^a^
Mode of delivery, *n* (%)				0.13
Vaginal delivery	20 (35.7)	12 (22.6)	Reference	
Cesarean	36 (64.3)	41 (77.4)	0.83 (0.65–1.06)	
Reason for C-section, *n* (%)				
Repeat C-sections	8 (17.4)	13 (26.5)	0.62 (0.28–1.37)	0.22
Fetal distress	8 (17.4)	10 (20.4)	0.80 (0.34–1.88)	0.60
Maternal disease	12 (26.1)	13 (26.5)	0.92 (0.46–1.85)	0.82
Cephalopelvic disproportion/macrosomia	3 (6.5)	4 (8.2)	0.75 (0.18–3.20)	1.00^b^
Failure to induce labor	8 (17.4)	3 (6.1)	2.67 (0.74–9.55)	0.11
Other	7 (15.2)	6 (12.3)	1.17 (0.42–3.26)	0.76
Maternal morbidity, *n* (%)				0.89^b^
No morbidity	48 (85.7)	44 (83)	Reference	
PE	7 (12.5)	8 (15.1)	0.86 (0.32–2.12)	
Other	1 (1.8)	1 (1.9)	0.92 (0.06–14.25)	
Maternal admission to the intensive unit care, *n* (%)	5 (9.1)	8 (15.1)	0.60 (0.21–1.72)	0.33

^a^Calculated with chi-square test; ^b^calculated with Fisher's exact test; PE: preeclampsia.

**Table 3:** Neonatal outcomes in pregnant women with chronic hypertension and/or previous PE, according to exercise or non-exercise group.

Outcome^d^	Study group *n* (%)	Control group *n* (%)	Relative risk (95% CI)	*P *value^a^
Birth weight, g (*n* = 108)				0.55^b^
<2500	9 (16.4)	11 (20.7)	0.83 (0.38–1.84)	
2500–3999	41 (74.5)	40 (75.5)	Reference	
≥4000	5 (9.1)	2 (3.8)	2.28 (0.47–11.14)	
Neonatal adequacy of weight to gestational age (*n* = 108)				0.45
SGA	5 (9.1)	9 (17)	0.53 (0.19–1.46)	
AGA	41 (74.5)	35 (66)	Reference	
LGA	9 (16.4)	9 (17)	0.88 (0.38–2.02)	
Gestational age at birth, wk (*n* = 108)				0.10
<37	11 (20)	18 (34)	0.59 (0.31–1.13)	
≥37	44 (80)	35 (66)	Reference	
Apgar 1 minute (*n* = 107)^c^				0.04
<7	10 (18.2)	3 (5.8)	3.15 (0.92–10.82)	
≥7	45 (81.8)	49 (94.2)	Reference	
Apgar 5 minutes (*n* = 107)^c^				0.24^b^
<7	3 (5.45)	0 (0)	Not calculated	
≥7	52 (94.55)	52 (100)		
Neonatal admission to Intensive Unit Care, (*n* = 107)^c^				0.82^b^
Yes	12 (22.2)	13 (24.5)	0.91 (0.46–1.80)	
No	42 (77.8)	40 (75.5)	Reference	
Neonatal morbidity (*n* = 100)^c^				0.40
Yes	16 (32)	20 (40)	0.80 (0.47–1.36)	
No	34 (68)	30 (60)	Reference	

^a^Calculated with chi-square test; ^b^calculated with Fisher's exact test, SGA: small for gestational age, ADA: adequate for gestational age, LGA: large for gestational age; ^c^the number of subjects changed due to lack of data to the variables; ^d^excluded one case of fetal death in second trimester.

**Table 4:** Multiple regression analysis adjusted with relative risk.

Variable^c^	Relative risk adjusted^a^ (95% CI)
Birth weight, g	
<2500	0.56 (0.26–1.25)
>2500	Reference
Birth weight^b^, g	
<4000	Reference
>4000	2.16 (0.41–11.37)
Neonatal adequacy of weight to gestational age	
SGA/LGA	1.17 (0.73–1.87)
ADA	Reference
Gestational age at birth, wk	
<37	0.53 (0.26–1.06)
≥37	Reference

^a^Ajusted for body mass index, race/color, number of gestation, chronic hypertension, and previous preeclampsia; ^b^it was not possible to be adjusted for chronic hypertension; SGA: small for gestational age, ADA: adequate for gestational age, LGA: large for gestational age; ^c^excluded one case of fetal death in second trimester.

## Abbreviations

PE: Preeclampsia
RCT: Randomized controlled trial
CH: Chronic hypertension
ICU: Intensive therapy units
SG: Study group
SB: Stationary bicycle
CG: Control group
bpm: Beats per minute
BMI: Body mass index
RR: Relative risk.
